# Confidence Intervals for Adaptive Trial Designs II: Case Study and Practical Guidance

**DOI:** 10.1002/sim.70202

**Published:** 2025-08-08

**Authors:** David S. Robertson, Thomas Burnett, Babak Choodari‐Oskooei, Munya Dimairo, Michael Grayling, Philip Pallmann, Thomas Jaki

**Affiliations:** ^1^ MRC Biostatistics Unit University of Cambridge Cambridge UK; ^2^ University of Bath Bath UK; ^3^ MRC Clinical Trials Unit at UCL London UK; ^4^ Sheffield Centre for Health and Related Research (SCHARR), University of Sheffield Sheffield UK; ^5^ Statistics and Decision Sciences Janssen R&D High Wycombe UK; ^6^ Centre for Trials Research Cardiff University Cardiff UK; ^7^ University of Regensburg Regensburg Germany

**Keywords:** adaptive design, bootstrap, conditional inference, coverage, estimation, group sequential, interim analyses

## Abstract

In adaptive clinical trials, the conventional confidence interval (CI) for a treatment effect is prone to undesirable properties such as undercoverage and potential inconsistency with the final hypothesis testing decision. Accordingly, as is stated in recent regulatory guidance on adaptive designs, there is the need for caution in the interpretation of CIs constructed during and after an adaptive clinical trial. However, it may be unclear which of the available CIs in the literature are preferable. This paper is the second in a two‐part series that explores CIs for adaptive trials. Part I provided a methodological review of approaches to construct CIs for adaptive designs. In this paper (Part II), we present an extended case study based around a two‐stage group sequential trial, including a comprehensive simulation study of the proposed CIs for this setting. This facilitates an expanded description of considerations around what makes for an effective CI procedure following an adaptive trial. We show that the CIs can have notably different properties. Finally, we propose a set of guidelines for researchers around the choice of CIs and the reporting of CIs following an adaptive design.

## 1 Introduction

Clinical trials are traditionally run in a fixed manner that does not allow for interim looks at the data within the trial itself. In contrast, an adaptive design (AD) allows for pre‐planned modifications to the course of the trial based on interim data analyses [1, 2]. This added controlled flexibility can lead to improved trial efficiency (e.g., in terms of sample size, time and cost) while still maintaining scientific rigor. ADs have seen increasing use in clinical trials in recent years, and in particular master protocols leveraging ADs are becoming increasingly popular [3, 4].

A wide variety of different types of AD have been proposed in the literature, including the following common broad classes according to the trial adaptations considered:

- Early trial stopping: *Group sequential designs* (*GSDs*) allow the trial to stop early at interim looks, for example, for efficacy or futility/lack of benefit.
- Treatment selection: *Multi‐arm multi‐stage* (*MAMS*) designs test multiple treatment options in parallel (typically against a common control arm), allowing the dropping of treatment arm(s) that are not performing (as) well and/or early stopping of arms for efficacy.
- Population selection: *Adaptive enrichment* designs allow the clinical (sub)population of interest to be selected (“enriched”) at interim looks, typically using pre‐defined patient subpopulations (e.g., based on biomarker information).
- Changing randomization probabilities: *Response‐adaptive randomization* (*RAR*) allows updates of the randomization probabilities based on patient responses, for example, to favor treatment arm(s) that are performing well [5].
- Changing trial sample size: *Sample size re‐estimation* allows the sample size of the trial to be adjusted, for example, based on interim conditional power calculations or interim blinded or unblinded estimates of parameters for sample size calculation.

Further educational material on all of these ADs can be found in Burnett et al. [6], Pallmann et al. [1], and the PANDA online resource [7].

Regardless of the type of AD, it remains crucial that the integrity and validity of the trial are maintained. Appropriate estimation of treatment effects is a key part of trial validity, which includes not only point estimates [8, 9] but also quantification of the uncertainty around the estimated treatment effects as given by *confidence intervals* (CIs). Intuitively, CIs capture this uncertainty by offering an interval that is expected to typically contain the unknown parameter of interest.

An important consideration in practice then is whether proposed methods to construct CIs have desirable properties. Most importantly, this relates to the CI having the desired coverage probability (i.e., the long‐run probability that the CI contains the true unknown treatment effect of interest). However, there are numerous other considerations, including the width of the CI (all else being equal, narrower CIs are preferred as they are more informative), whether the CI will always contain an associated point estimate, and whether the CI will always be consistent with the decision rule (i.e., with an associated hypothesis test). The fundamental problem for ADs is that the use of standard CI methodology (i.e., CIs constructed using methods that do not account for the fact an AD has been used) may not necessarily result in desirable properties.

Recent regulatory guidance highlights these concerns, with the US Food and Drug Administration (FDA) noting that “confidence intervals for the primary and secondary endpoints may not have correct coverage probabilities for the true treatment effects” and thus “confidence intervals should be presented with appropriate cautions regarding their interpretation” [10]. The same guidance also highlights the need to pre‐specify methods used to compute CIs after an AD, see also the Adaptive designs CONSORT Extension (ACE) guidance [2, 11]. Meanwhile, the European Medicines Agency (EMA) guidance states that “methods to … provide confidence intervals with pre‐specified coverage probability are required” if an AD is used in a regulated setting [12].

These concerns and regulatory guidance motivate the growing body of literature proposing “adjusted” CIs that account for trial adaptations as per a particular trial design. However, in our experience there is at best limited uptake of adjusted CIs in practice (with the possible exception of “repeated” CIs (RCIs) in GSDs; see Part I of the paper series [13] and Section 3.1 of this current paper for a definition), with many adaptive trials continuing to only report the standard CI. Evidence for this in the context of two‐stage single‐arm trials can be found in [14], where only 2% of 425 articles reported an adjusted CI. This limited uptake of adjusted CIs for ADs is due to a number of reasons, including the lack of awareness of methods in the literature, available software/code, and guidance around the choice of adjusted CI in practice.

This paper is the second in a two‐part series that explores the issue of CIs for ADs. In Part I of the series, we reviewed and compared methods for constructing CIs for different classes of ADs and critically discussed different approaches. In the current paper (Part II), we consider CIs for ADs from a practical perspective and propose a set of guidelines for researchers around the choice and reporting of CIs following an AD. We first briefly describe performance measures of CIs in Section 2. We introduce the MUSEC trial case study in Section 3, which was a two‐stage GSD. This was chosen in order to illustrate the widest range of different types of adjusted CIs and for continuity with its use as a case study for the previously published point estimation for ADs guidance paper [9]. We show how to calculate different types of CIs (with R code provided). We use the case study as a basis for a simulation study in Section 4. We conclude with guidance for researchers and discussion in Sections 5 and 6, respectively.

## 2 Performance Measures for CIs

As introduced in Part I of this paper series, different desirable properties for CIs have been proposed, which we recapitulate below. To fix ideas, suppose we have a random sample X from a probability distribution with parameter θ, which is the single parameter of interest in the trial. A CI for θ with confidence level 1−α is a random interval (L(X),U(X)) that has the following (claimed) property: P(L(X)<θ<U(X))=1−α for all θ.

The *coverage* probability (often shortened to just “coverage”) of a CI (L(X),U(X)) is given by P(L(X)<θ<U(X)). This is the key performance measure for a CI, given that the definition itself of a CI is based around actually having the claimed “nominal” 1−α confidence level.

Alongside coverage, other criteria for CIs (generally, and specifically for ADs) have been proposed in the literature. The main criteria/performance measures include:

- Correct coverage (arguably essential).
- Width (all other things being equal, a smaller width is desirable).
- Consistency/compatibility with the hypothesis test (see below).
- Contains the point estimate of interest.
- (Approximate) symmetry around the point estimate of interest.
- Is in fact an interval (i.e., not a union of disjoint intervals, or the empty set).
- Is computationally feasible/simple to implement.

A CI is *consistent*/*compatible* with the hypothesis testing decision if it excludes the parameter value(s) that are rejected by the hypothesis test, and conversely includes the parameter value(s) that are *not* rejected by the hypothesis test. If a CI is *not* consistent/compatible with the hypothesis testing decision, then this can lead to problems with study interpretation and the communication of results.

## 3 Case Study

In this section, we illustrate how different types of adjusted CIs (as reviewed in Part I of this paper series [13]) can be used in practice for a GSD. We use the MUSEC (MUltiple Sclerosis and Extract of Cannabis) trial [15, 16] as our case study, which assessed the effect of oral cannabis extract (CE) on muscle stiffness in adults with stable multiple sclerosis compared to placebo. The primary outcome was binary—whether or not a patient had “relief from muscle stiffness” after 12 weeks of treatments, based on a dichotomized 11‐point category rating scale. MUSEC utilized a two‐stage GSD, with early stopping for superiority only (i.e., no early stopping for futility) assessed using an O'Brien–Fleming (OBF) boundary. The unblinded interim analysis was planned after 200 participants (100 per arm) had completed the 12‐week treatment course, with the final analysis planned after 400 patients (200 per arm) if the interim stopping rule was not met.

As mentioned in Section 1, we use a GSD as our case study in order to illustrate the widest range of different classes of adjusted CIs (both unconditional and conditional). In addition, we use the MUSEC trial for continuity with the case study accompanying a previously published review of point estimation for ADs [9].

The clinical context of the MUSEC trial helps justify the use of a GSD with early stopping for efficacy but not for lack of benefit (futility). A previous trial had already demonstrated a (small) significant effect of using cannabinoids on muscle spasticity, but using a single‐item semi‐objective rating scale. Part of the motivation of the MUSEC trial was to use a more up‐to‐date and patient‐oriented measure of efficacy. Given the previous trial results, it is plausible that stopping early for futility was deemed unlikely; hence, the focus on stopping early for efficacy in order to reduce the required time and sample size required under the envisaged effect size based on the results of the previous trial. However, as pointed out by an anonymous reviewer, allowing early termination for efficacy without allowing for a (binding) futility decision requires more stringent criteria for the efficacy decision to prevent inflation of the Type I error rate, which in turn requires a larger maximal sample size to maintain power.

Another feature of the design is the use of OBF boundaries, which are conservative in the sense that it is more difficult to stop early at the earlier analyses. Given the standardized test statistics *Z*_*k*_ for group *k* = 1, …, *K*, the one‐sided OBF boundaries and stopping rules take the following form [17]:

After group *k* = 1, …, *K* − 1

$$\text{if}\;Z_{k}\geq C\left(K,\alpha \right)\surd \left(K/k\right)\;\text{stop},\text{reject}\;H_{0}$$

$$\begin{array}{cc} \;\text{otherwise} & \;\text{continue to group}\;k+1 \end{array}$$

After group *K*

$$\begin{array}{cc} \text{if}\;Z_{K}\geq C\left(K,\alpha \right) & \;\text{stop},\text{reject}\;H_{0} \end{array}$$

$$\begin{array}{cc} \;\text{otherwise} & \;\text{stop},\mathrm{do}\;\text{not reject}\;H_{0} \end{array}$$

Here, the values of C(K,α) are chosen to ensure that the overall Type I error probability for the *K‐*stage trial is controlled at preset level *α*. These can be calculated using classical group sequential theory based on independent increments [18] (i.e., the test statistic calculated using the data gathered in a new stage of a trial is statistically independent of the information gathered in all previous stages), where the independent increments property holds because of randomization of patients to treatments and the choice of endpoint.

In the actual trial, an unblinded sample size re‐estimation based on conditional power considerations [15] was also planned and carried out at the interim analysis, which reduced the total planned sample size from 400 to 300. For the purpose of illustrating the calculation of a larger range of adjusted CIs, we ignore this sample size re‐estimation in what follows. If we were to take this into account, then the methods for adaptive GSDs would apply (see Section 4 of Part I of this paper series [13]), but fewer classes of adjusted CIs are available in that setting. Wassmer and Brannath [19] report the adjusted CIs (RCIs and final unconditional CIs) available for this trial taking into account the sample size re‐estimation. For more general guidelines around best practice in this context, see Section 5 of this paper. We note in passing that the risk of operational bias due to the unblinding of the interim data was mitigated by having an Independent Data Monitoring Board.

Ultimately, the trial did continue to its second stage; Table 1 summarizes the study data at the interim and final analyses, as well as the OBF efficacy stopping boundaries. The parameter of interest is $\theta =p_{\mathrm{CE}}-p_{P}$, which is the difference in the response rates in the CE and placebo arms, with corresponding test of the null hypothesis $H_{0}\;:\;\theta =0$ versus the alternative $H_{1}\;:\;\theta >0$.

**TABLE 1:** MUSEC trial observed data, by analysis stage.

	Interim analysis		Final analysis	
	Placebo	Cannabis extract	Placebo	Cannabis extract
Number of patients with relief from muscle stiffness	12	27	21	42
Total number of subjects	97	101	134	143
Observed standardized test statistic	2.540		2.718	
O'Brien–Fleming stopping boundary for efficacy	2.797		1.977	
O'Brien–Fleming stopping boundary for futility	−∞		1.977	

*Note:* The O'Brien–Fleming stopping boundaries are also shown.

As can be seen, at the interim analysis the standardized test statistic was close to the stopping boundary for early rejection of the null hypothesis (no difference in the proportion of subjects with relief from muscle stiffness between treatment arms).

Typically, at the final analysis a 100(1−α)%CI will be desired for the parameter of interest $=p_{\mathrm{CE}}-p_{P}$. A common method of achieving this is to use a standard two‐sided interval, for example, based on Wald's methodology [20]. For MUSEC, denoting the final sample sizes in the two arms by $n_{P}=134$ and $n_{\mathrm{CE}}=143$, and the MLEs for the rates by ${\hat{p}}_{P}=21/n_{P}$ and ${\hat{p}}_{\mathrm{CE}}=42/n_{\mathrm{CE}}$, this gives for α=0.05:

$$\begin{array}{cc} & \left({\hat{p}}_{\mathrm{CE}}-{\hat{p}}_{P}\right)\pm \Phi^{-1}\left(1-\alpha /2\right)\hat{\mathrm{Var}}\left({\hat{p}}_{\mathrm{CE}}-{\hat{p}}_{P}\right)\\ & \;=\left({\hat{p}}_{\mathrm{CE}}-{\hat{p}}_{P}\right)\pm \Phi^{-1}\left(1-\alpha /2\right)\sqrt{\frac{{\hat{p}}_{\mathrm{CE}}\left(1-{\hat{p}}_{\mathrm{CE}}\right)}{n_{\mathrm{CE}}}+\frac{{\hat{p}}_{P}\left(1-{\hat{p}}_{P}\right)}{n_{P}}}\\ & \;=\left(0.040,0.234\right) \end{array}$$

where $\Phi^{-1}\left(\cdot \right)$ denotes the inverse cumulative distribution function (CDF) of a standard normal random variable.

If the interim analysis was not present, this CI would have a number of desirable properties (as discussed in Section 2): it would be guaranteed to be an interval containing the MLE for ${\hat{p}}_{\mathrm{CE}}-{\hat{p}}_{P}$, would (at least asymptotically) have the desired coverage, would be consistent with the associated hypothesis test, and would evidently be easily computed.

In this section (as well as the simulation study in Section 4), we illustrate how different types of CIs (both unconditional and conditional) can be used in practice for a GSD, based on the MUSEC trial. We use a GSD as our case study in order to illustrate the widest range of different CIs.

### 3.1 Calculation of CIs When Continuing to Stage 2

Using the observed data from the MUSEC trial, we now show how to calculate various CIs for the treatment difference, denoted $\theta =p_{\mathrm{CE}}-p_{P}$, from both a conditional and unconditional perspective (see explanation on the difference below) when the trial continues to Stage 2, as happened for the MUSEC trial. R code to obtain all CIs below is provided as detailed in the Data Availability statement.

As already seen above, the *standard*/*naive CI* for the treatment difference (i.e., the Wald CI) is given by

$$\begin{array}{cc} \hat{\theta }\pm \Phi^{-1}\left(1-\alpha /2\right)\hat{\mathrm{Var}}\left(\hat{\theta }\right) & =\left({\hat{p}}_{\mathrm{CE}}-{\hat{p}}_{P}\right)\pm \Phi^{-1}\left(1-\alpha /2\right)\\ & \;\times \sqrt{{\hat{p}}_{\mathrm{CE}}\left(1-{\hat{p}}_{\mathrm{CE}}\right)/n_{\mathrm{CE}}+{\hat{p}}_{P}\left(1-{\hat{p}}_{P}\right)/n_{p}} \end{array}$$

Other methods than Wald are available to calculate standard/naive CIs for the difference of two proportions [21, 22], but none of these will be able to account for the AD used.

From an unconditional perspective, we want to estimate *θ* regardless of the stage that the trial stops, and are interested in the properties of the CI as averaged over all possible stopping times (weighted by the respective stage‐wise stopping probabilities). Note that the standard/naive CI above is an unconditional CI. In contrast, from a conditional perspective, we are interested in estimation conditional on the stage the trial stops at (so for the MUSEC trial, conditional on the trial continuing to Stage 2).

In what follows, we let *e*_1_, *e*_2_ denote the efficacy stopping boundaries, $I_{1},I_{2}$ the (observed) information, at Stages 1 and 2, respectively, and *T* the stage the trial stopped at (so *T* = 2 for the MUSEC trial). The definitions of the information $I_{1}$ and $I_{2}$ for the MUSEC trial are given in Appendix 1, which depend on the number of observed successes.

#### 3.1.1 Unconditional CIs

The *final unconditional CI* (also known as the exact unconditional CI) depends on a choice of the ordering of the sample space with respect to evidence against the null hypothesis. In what follows, we use stagewise ordering, which has desirable properties described by Jennison and Turnbull [17]. This allows the use of the *p*‐value function P(θ) to find the lower and upper bounds for the 95% CI, ${\hat{\theta }}_{l}$ and ${\hat{\theta }}_{u}$, which are the solutions to the equations *P*(${\hat{\theta }}_{l}$) = 0.025 and *P*(${\hat{\theta }}_{u}$) = 0.975. The formula for the *p*‐value function for stopping stage *T* = 2 (with observed second‐stage test statistic $Z_{2}=z_{2}$, where $Z_{2}=\hat{\theta }\sqrt{I_{2}}$) is given in Appendix 1. Note that the associated point estimator of this method, the median unbiased estimator (MUE), ${\hat{\theta }}_{\mathrm{MUE}}$, is the solution to the equation *P*(${\hat{\theta }}_{\mathrm{MUE}}$) = 0.5. If the distributional assumptions hold exactly (i.e., the joint canonical distribution of test statistics holds exactly), then the final unconditional CI guarantees consistency with the test decision. Software for computing this CI in R for general GSDs can be found in the PANDA resource [7].

The *RCI* follows a simple form: $\hat{\theta }\pm e_{T}/\sqrt{I_{T}}$, see [17]. Note that there is no explicit associated point estimator with this method (although one could of course just use the standard MLE $\hat{\theta }$). Since $\hat{\theta }=Z_{T}/\sqrt{I_{T}}$, the RCI guarantees consistency with the test decision. Software for computing RCIs in R for general GSDs can be found in the PANDA resource [7].

The *adjusted asymptotic CI* adjusts the standard CI, giving a CI of the form

$$\left(\hat{\theta }-\hat{\mu }\left(\hat{\theta }\right)\right)\pm \Phi^{-1}\left(1-\alpha /2\right)\hat{\sigma }\left(\hat{\theta }\right)/\sqrt{I_{T}}$$

where $\hat{\mu }\left(\hat{\theta }\right)$ and $\hat{\sigma }\left(\hat{\theta }\right)$ are functions of $\hat{\theta }$ given in [23] and reproduced in Appendix 1. The associated point estimator for this method is the bias‐adjusted estimator $\hat{\theta }-\hat{\mu }\left(\hat{\theta }\right)$.

One subtlety with the use of the adjusted asymptotic CI (for trials that continue to Stage 2) is that it is possible for the information levels to actually *decrease* from Stages 1 to 2, that is, $I_{2}<I_{1}$. This can happen in trials with a binary outcome when the pooled estimated response rate is very close to zero (or 1) at Stage 1, but further away from zero (and 1) at Stage 2. In this situation, it is not possible to calculate the adjusted asymptotic CI, although this happens very rarely (i.e., one or two times out of $10^{5}$ simulation replicates) for trials with success rates similar to those observed in the MUSEC trial. It is similarly possible for this to occur in trials with, for example, normal (if the estimated standard deviation differs greatly between stages) and time‐to‐event data (typically when the number of events that occur between analyses is smaller), though again is a rare occurrence.

For the *parametric bootstrap CI*, we use a simple bootstrap algorithm to generate *B* bootstrap MLEs ${\hat{\theta }}^{\left(b\right)}$ for *b* = 1, …, *B*. In the interests of space, we defer the details to Appendix 1. Note that the associated point estimator for this method is given by the mean of ${\hat{\theta }}^{\left(1\right)},\;\ldots ,{\hat{\theta }}^{\left(B\right)}$.

Finally, the *randomization‐based CI* follows a different bootstrap procedure in order to calculate an adjusted *p*‐value, based on the randomization distribution (this being an example of randomization‐based inference, i.e., a randomization test). The idea is to reproduce the group sequential analysis for each allowable allocation of patients to treatments, but *keeping the observed patient outcomes fixed*. The adjusted *p*‐value is then the proportion of these potential results that are as extreme or more extreme than the actual trial result. Note that at the interim analysis, exactly the same set of patients is used each time (although with different treatment allocations). We give more details on the procedure as detailed in [24] in Appendix 1.

In practice, it is not feasible (except for small sample sizes) to use the entire set of possible random allocations and we use *N* random samples from the set instead. However, a problem arises when the adjusted *p*‐value is equal to zero, that is, no allocations are sampled that give results as extreme as or more extreme than those actually observed. Even when *N* = 10^4^ this can occur as discussed later in the simulation study.

#### 3.1.2 Conditional CIs

The *final conditional CI* (also known as the exact conditional CI) uses the conditional density of the MLE, and is defined as:

$$CI_{c}=\left\{\theta :\alpha /2<\text{Prob}\left(\hat{\theta }\geq {\hat{\theta }}_{\mathrm{obs}}\;|\;T=t,\theta \right)<1-\alpha /2\right\}$$

where ${\hat{\theta }}_{\mathrm{obs}}$ is the observed value of the MLE, see [25], and further details provided in Appendix 1. The associated point estimator is the conditional MUE, ${\hat{\theta }}_{\text{CMUE}}$, which is the solution to the following equation:

$$\text{Prob}\left(\hat{\theta }\geq {\hat{\theta }}_{\mathrm{obs}}\;|\;T=t,\theta \right)=0.5$$

Like for the adjusted asymptotic CI, if the information levels decrease from Stages 1 to 2, that is, $I_{2}<I_{1}$, then the final conditional CI (and hence the restricted final conditional CI, see below) cannot be calculated.

The *restricted final conditional CI* (also known as the restricted exact conditional CI), as the name suggests, restricts the range of the final conditional CI. Given the final conditional CI ($CI_{c}$) defined above, the restricted final conditional CI is defined to be $I_{c}\cap CI_{r}$, where:

$${\mathrm{CI}}_{r}=\left\{\theta :\text{Prob}\left(T\leq t\;|\;\theta \right)>\alpha ⁄2\;\text{and}\;\text{Prob}\left(T\geq t\;|\;\theta \right)>\alpha ⁄2\right\}$$

See [25]. For a trial continuing to Stage 2 (so T=2), the upper bound of the restricted final conditional CI is set equal to $\min\left\{{\hat{\theta }}_{u},\left(e_{1}-\Phi^{-1}\left(\alpha ⁄2\right)\right)⁄\sqrt{I_{1}}\right\}$, where ${\hat{\theta }}_{u}$ is the upper bound of $CI_{c}$. Note that if this minimum is less than the upper bound of $CI_{c}$, the intersection $CI_{c}\cap CI_{r}$ is empty and the restricted final conditional CI is the empty set.

The *conditional likelihood CI* is based on the conditional MLE, that is, the maximizer of the conditional log‐likelihood. As shown in [26], the conditional log‐likelihood, conditioning on the trial stopping at stage *T* = *t* is given by

$$L_{c}\left(\theta ,z_{t};t\right)=-\frac{1}{2}{\left(z_{t}-\theta \sqrt{I_{t}}\;\right)}^{2}-\log\left(\text{Prob}\left(T=t\;|\;\theta \right)\right)$$

Using the results of Fan and DeMets [25], we can show that the conditional MLE, ${\hat{\theta }}_{c}$, is the solution of the following equation when *T* = 2:

$${\hat{\theta }}_{c}={\hat{\theta }}_{\mathrm{obs}}-\frac{\sqrt{I_{1}}\;\phi \left(e_{1}-{\hat{\theta }}_{c}\;\sqrt{I_{1}}\right)}{I_{2}\Phi \left(e_{1}-{\hat{\theta }}_{c}\;\sqrt{I_{1}}\right)}$$

where ϕ and Φ are the probability density function (PDF) and CDF of the standard normal distribution, respectively. The conditional MLE is the associated point estimator for the conditional likelihood CI. The conditional likelihood CI is calculated using a conditional bootstrap procedure, with full details provided in Appendix 1.

The *penalized likelihood CI* is equal to the conditional likelihood CI when the trial continues to Stage 2, see Section 3.2 for details of how it is different when in fact the trial stops at Stage 1.

### 3.2 Calculation of CIs When Stopping Early

In this subsection, we detail how the various CIs are calculated when the stopping stage is *T* = 1 (rather than *T* = 2 as in Section 3.1), which will be needed for the Simulation study in Section 4. Again, code to calculate all CIs is provided below.

The *standard*/*naive CI* for the treatment difference (i.e., the Wald CI) is given by

$$\begin{array}{cc} \hat{\theta }\pm \Phi \left(1-\alpha /2\right)\hat{\mathrm{Var}}\left(\hat{\theta }\right) & =\left({\hat{p}}_{1,\mathrm{CE}}-{\hat{p}}_{1,P}\right)\pm \Phi \left(1-\alpha /2\right)\\ & \;\times \sqrt{\frac{{\hat{p}}_{1,\mathrm{CE}}\left(1-{\hat{p}}_{1,\mathrm{CE}}\right)}{n_{1,\mathrm{CE}}}+\frac{{\hat{p}}_{1,P}\left(1-{\hat{p}}_{1,P}\right)}{n_{1,P}}} \end{array}$$

where ${\hat{p}}_{1,P}$ and ${\hat{p}}_{1,\mathrm{CE}}$ are the mean response rates in Stage 1 for the placebo and CE arms, respectively, while $n_{1,P}$ and $n_{1,\mathrm{CE}}$ are the number of patients allocated in Stage 1 to the placebo and CE, respectively.

#### 3.2.1 Unconditional CIs

For the *final unconditional CI*, we again use the *p*‐value function P(θ) (based on stagewise ordering of the sample space) to find the lower and upper and bounds for the 95% CI, ${\hat{\theta }}_{l}$ and ${\hat{\theta }}_{u}$, which are the solutions to the equations *P*(${\hat{\theta }}_{l}$) = 0.025 and *P*(${\hat{\theta }}_{u}$) = 0.975. This admits a closed‐form expression when *T* = 1, with ${\hat{\theta }}_{l}=\frac{\Phi^{-1}\left(\alpha ⁄2\right)+Z_{1}}{\sqrt{I_{1}}}$ and ${\hat{\theta }}_{u}=\frac{\Phi^{-1}\left(1-\alpha ⁄2\right)+Z_{1}}{\sqrt{I_{1}}}$ where $Z_{1}=\hat{\theta }\sqrt{I_{1}}$.

The *RCI* and *adjusted asymptotic CI* are given in Section 3.1, since they are written in terms of a general stopping stage *T*.

The *parametric bootstrap CI* procedure is the same as before, except that now ${\hat{p}}_{\mathrm{CE}}$ and ${\hat{p}}_{P}$ represent the Stage 1 response rate estimates on the CE and placebo arm, respectively.

#### 3.2.2 Conditional CIs

When *T* = 1, the *final conditional CI* has the same definition as given for $CI_{c}$ in Section 3.1. More explicitly, for a trial stopping at Stage 1, the lower and upper bounds for the CI, denoted ${\hat{\theta }}_{l}$ and ${\hat{\theta }}_{u}$, are the solutions to the equations $\alpha ⁄2=\frac{1-\Phi \left(\sqrt{I_{1}}\left[{\hat{\theta }}_{\mathrm{obs}}-{\hat{\theta }}_{l}\right]\right)}{1-\Phi \left(e_{1}-\sqrt{I_{1}}{\hat{\theta }}_{l}\;\right)}$ and $1-\alpha ⁄2=\frac{1-\Phi \left(\sqrt{I_{1}}\left[{\hat{\theta }}_{\mathrm{obs}}-{\hat{\theta }}_{u}\right]\right)}{1-\Phi \left(e_{1}-\sqrt{I_{1}}{\hat{\theta }}_{u}\;\right)}$. Note that as the trial stops closer to the boundary, the final conditional CI has increasingly poor properties, with the lower bound becoming increasingly negative. In extreme situations, the lower bound can even go below −1 which is nonsensical given that $p_{\mathrm{CE}}-p_{P}\in \left[-1,1\right]$. In such a case, one option is to truncate the CI to have a lower bound equal to −1.

Similarly, the *restricted final conditional CI* has the same definition $CI_{c}\cap CI_{r}$ with $CI_{r}$ as given in Section 3.1. For a trial stopping at Stage 1, the lower bound of the restricted final conditional CI is set equal to $\max\left\{{\hat{\theta }}_{l},\left(e_{1}-\Phi^{-1}\left(1-\alpha ⁄2\right)\right)⁄\sqrt{I_{1}}\right\}$, where ${\hat{\theta }}_{l}$ is the lower bound of $CI_{c}$. Note that if this maximum is greater than the upper bound of $CI_{c}$, the intersection $CI_{c}\cap CI_{r}$ is empty and the restricted final conditional CI is the empty set.

The *conditional likelihood CI* is based on the conditional log‐likelihood as given in Section 3.1. Again using the results of Fan and DeMets [25], the conditional MLE, ${\hat{\theta }}_{c}$, is the solution of the following equation when *T* = 1:

$${\hat{\theta }}_{c}={\hat{\theta }}_{\mathrm{obs}}-\frac{\phi \left(e_{1}-{\hat{\theta }}_{c}\;\sqrt{I_{1}}\right)}{\sqrt{I_{1}}\Phi \left(e_{1}-{\hat{\theta }}_{c}\;\sqrt{I_{1}}\right)}$$

The conditional likelihood CI is calculated using a conditional bootstrap procedure, with full details given in Appendix 1. Like for the final conditional CI, this CI also has increasingly poor properties as the trial data becomes closer to the boundary, and the lower bound can also go below −1.

Finally, the *penalized likelihood CI* is different from the conditional likelihood CI when the trial stops at Stage 1. As described in [26], the penalized log‐likelihood is given by $L_{\lambda }\left(\theta ;z_{t},t\right)=-\frac{1}{2}{\left(z_{t}-\theta \sqrt{I_{t}}\;\right)}^{2}-\lambda \;\log\;\text{Prob}\left(T=t\;|\;\theta \right)$. Hence the MLE and the conditional MLE correspond to maximizing the penalized log‐likelihood when λ=0 and λ=1, respectively. For a given choice of λ∈[0,1] this gives a penalized likelihood estimate $P\left(\lambda ,z_{t},t\right)=\text{argma}x_{\theta }\;L_{\lambda }\left(\theta ;z_{t},t\right)$. The choice of λ proposed is $\lambda^{*}=\left\{\lambda \in \left[0,1\right]\;:\;P\left(\lambda ,e_{1},1\right)=0\right\}$. With this choice, the penalized MLE is defined as ${\hat{\theta }}_{p}=P(\lambda^{*},z_{1},1)$. The penalized likelihood CI is then calculated using the same bootstrap procedure as above for the conditional likelihood CI, with the bootstrap conditional MLE ${\hat{\theta }}_{c}^{\left(b\right)}$ replaced by the penalized MLE ${\hat{\theta }}_{p}^{\left(b\right)}$. Note that by conditioning the bootstrap sampling on early stopping, each bootstrap replication satisfies ${\hat{\theta }}_{p}^{\left(b\right)}>0$, which therefore guarantees that the associated CI lies above zero, consistent with the decision to stop the study early for benefit.

### 3.3 Results From the MUSEC Trial

**FIGURE A1:**
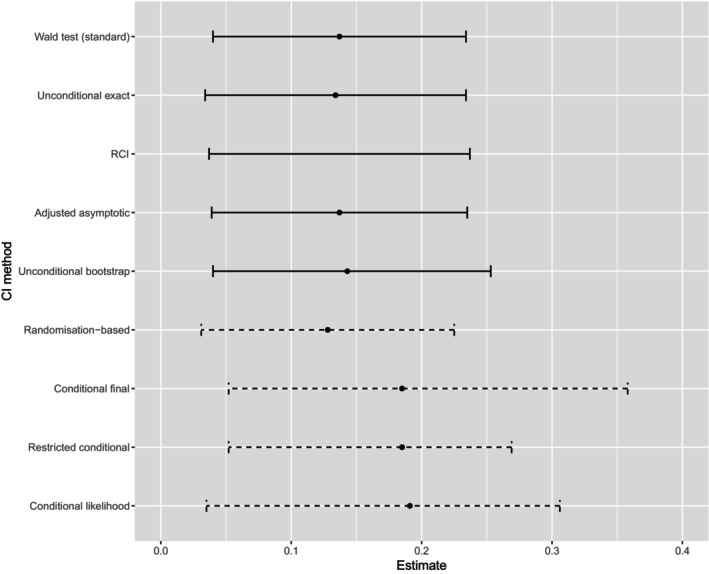
Graphical representation of confidence intervals (and associated point estimates) calculated using the observed data from the MUSEC trial. Dashed lines indicate conditional CIs. RCI, repeated confidence interval.

Table 2 gives the values of all the CIs described in Section 3.1, calculated using the observed data and OBF stopping boundaries from the MUSEC trial, with Figure A1 in Appendix 2 giving the graphical representation. When there is an associated point estimate (as described above in Section 2.1), this is also shown. For the methods requiring repeated sampling/simulation (i.e., the unconditional parametric bootstrap CI, unconditional randomization‐based CI and conditional (penalized) likelihood CI), we use *N* = 10^6^ trial replicates.

**TABLE 2:** Confidence intervals (and associated point estimates) calculated using the observed data and O'Brien–Fleming efficacy stopping boundaries from the MUSEC trial.

Type of CI	CI method	Point estimate	95% two‐sided CI	CI width
Standard/naive	Wald test	0.137 (overall MLE)	(0.040, 0.234)	0.194
Unconditional	Final	0.134 (MUE)	(0.034, 0.234)	0.200
	Repeated	—	(0.037, 0.237)	0.199
	Adjusted asymptotic	0.137	(0.039, 0.235)	0.196
	Parametric bootstrap	0.143	(0.041, 0.253)	0.212
	Randomization based	0.130	(0.033, 0.226)	0.194
Conditional	Final	0.185 (conditional MUE)	(0.052, 0.358)	0.306
	Restricted final	0.185 (conditional MUE)	(0.052, 0.269)	0.217
	(Penalized) likelihood	0.191 (conditional MLE)	(0.034, 0.304)	0.271

The Wald (standard) CI is (0.040, 0.234) with a width of 0.194, and is the comparator for all the other CIs in Table 2, since it is the conventional end‐of‐trial CI. Starting with the unconditional CIs, all are similar to the standard CI. In contrast, the conditional CIs all are substantially wider than the standard CI, with an increase of 58%, 12%, and 40% for the conditional final, restricted final, and (penalized) likelihood CIs, respectively. This reflects the loss of information associated with conditioning on the stopping stage *T* = 2. The conditional point estimators are also substantially higher than the unconditional point estimators. This upward correction is intuitive from a conditional perspective: there is downward “selection pressure” on the MLE calculated at the end of Stage 1, since if this is large then the trial does not continue to Stage 2. For the conditional CIs, this is also reflected in their upper confidence limits being substantially higher compared with the standard CI. Finally, there is asymmetry in the restricted final CI around its associated point estimate, reflecting how the upper confidence limit of the conditional final CI is adjusted.

As a sensitivity analysis to further demonstrate the potential differences between the adjusted CIs, we follow Emerson et al. [27] and calculate the adjusted CIs (and associated point estimates) at the group sequential stopping boundaries, that is, when $Z_{1}\approx e_{1}$ and the trial stops at Stage 1, and when $Z_{2}\approx e_{2}$ and the trial continues to Stage 2 (the equalities here are approximate since we start with assumed values of the underlying binary data). The tables of results at both stopping boundaries can be found in Appendix 2.

When $Z_{1}\approx e_{1}$ and the trial stops at Stage 1, the unconditional CIs are similar to the standard CI, with the exception of the RCI which is substantially wider (CI width of 0.391 vs. 0.268). All of the conditional CIs, with the exception of the penalized likelihood CI, have very poor properties—the final conditional CI and conditional likelihood CI have a lower bound substantially lower than −1, while the restricted final conditional CI is the empty set. This is consistent with the literature [25, 26] that does not recommend the use of these two methods when a group sequential trial stops early for efficacy. Intuitively, these results are due to the observed data being “extreme” conditionally as $Z_{1}$ is very close to $e_{1}$. The penalized likelihood CI is much more sensible with a width of 0.336, which is less than the width of the RCI, but does have an associated point estimate that is outside the CI.

When $Z_{2}\approx e_{2}$, the general pattern of results is similar to those in Table 2 based on the data actually observed in the MUSEC trial. The unconditional CIs are similar to the standard CIs, with the conditional CIs being wider. Reassuringly, all of the CIs have a lower bound (approximately) equal to 0, reflecting how $Z_{2}$ is (approximately) equal to the stopping boundary. This time, the restricted final CI does not have a marked asymmetry and is equal to the conditional final CI.

For the MUSEC trial design, the use of different methods can give noticeably different CIs for the treatment effect, particularly when considering a conditional versus unconditional perspective. This could influence the interpretation of the trial results, and highlights the importance of pre‐specifying which CI(s) will be reported following an AD. The choice of CI(s) will depend on what the researchers wish to achieve regarding the estimand [28] in question. There will be pros and cons for the different CI methods, which we explore further in the simulation study in Section 4. We also note that there is a strong link between design and estimation—the CIs above depend on the design of the trial, and would potentially change with different group sequential stopping boundaries.

## 4 Simulation Study

### 4.1 Simulation Set‐Up

Since the CIs calculated above are one realization of the trial data given the trial design, in this section we carry out a simulation study to investigate the performance of the CIs under different scenarios. Note that we have *not* used assumed values for the underlying treatment effects to calculate the CIs given in Table 2. As can be seen from the formulae in Section 3.1, these CIs only depend on the observed data and efficacy stopping boundary.

To demonstrate the properties of the CIs when averaged over many trial realizations following the two‐stage design of the MUSEC trial, we ran simulations under different values of $p_{\mathrm{CE}}$, the assumed true value of the response rate for the CE arm. For simplicity, we kept the value of $p_{P}$, the assumed true value of the response rate for the placebo arm, equal to the value observed in the MUSEC trial, that is, $p_{P}=\hat{p_{p}}=21/134\approx 0.157$. We also keep the value of $e_{1}$, the efficacy stopping boundary, equal to 2.797 as used in the MUSEC trial. Note that by keeping the value of $e_{1}$ fixed, the efficiency of the trial will increase (i.e., the expected sample size will decrease) as the effect size $p_{\mathrm{CE}}-p_{P}$ increases, since there is a greater chance of early stopping for efficacy.

We used the following procedure for our trial simulations:

1. Given the assumed value for $p_{\mathrm{CE}}$, denoted $p_{\mathrm{CE}}^{*}$, generate *N* Stage 1 trial replicates $S_{1,\mathrm{CE}}^{\left(1\right)},\ldots ,S_{1,\mathrm{CE}}^{\left(N\right)}$ and $S_{1,P}^{\left(1\right)},\ldots ,S_{1,P}^{\left(N\right)}$, where $S_{1,\mathrm{CE}}^{\left(i\right)}˜\mathrm{Bin}\left(n_{1,\mathrm{CE}},p_{\mathrm{CE}}^{*}\right)$ and $S_{1,P}^{\left(i\right)}˜\mathrm{Bin}\left(n_{1,P},{\hat{p}}_{P}\right)$.
2. For *i* = 1, …, *N* calculate the standardized Stage 1 test statistic $Z_{1}^{\left(i\right)}$ from the bootstrap values $S_{1,\mathrm{CE}}^{\left(i\right)}$ and $S_{1,P}^{\left(i\right)}$.
  - If $Z_{1}^{\left(i\right)}>e_{1}$ then calculate all CIs (conditional and unconditional) with *T* = 1.
  - Otherwise, generate a Stage 2 trial replicate $S_{2,\mathrm{CE}}^{\left(i\right)}$ and $S_{2,P}^{\left(i\right)}$, where $S_{2,\mathrm{CE}}^{\left(i\right)}˜\mathrm{Bin}\left(n_{\mathrm{CE}}-n_{1,\mathrm{CE}},p_{\mathrm{CE}}^{*}\right)$ and $S_{2,P}^{\left(i\right)}˜\mathrm{Bin}\left(n_{p}-n_{1,P},{\hat{p}}_{P}\right)$. Then calculate all CIs (conditional and unconditional) with *T* = 2.

For each value of $p_{\mathrm{CE}}$, we simulated *N* = 10^5^ trial replicates. For the CI methods requiring a bootstrap procedure, we used *B* = 10^4^ bootstrap samples. For the randomization‐based CI, even with 10^4^ samples we still frequently ran into the issue of $p_{\text{adjusted}}=0$. Hence, we did not consider this CI method further in the simulation study.

For each of the CI methods considered, we evaluated the following properties:

1. Coverage.
2. Mean CI width and standard deviation (SD) of CI width.
3. Consistency.
4. Lower coverage: probability that the lower confidence limit is above the true value of θ, denoted P(L(X)>θ).
5. Upper coverage: probability that the upper confidence limit is below the true value of θ, denoted P(U(X)<θ).

### 4.2 Simulation Results

#### 4.2.1 Overall (Unconditional) Results

Table 3 shows the overall (unconditional) simulation results with the true success rates $\left(p_{p},p_{\mathrm{CE}}\right)$ on the two arms equal to the overall observed means in the MUSEC trial, that is, $p_{p}=21/134\approx 0.157$ and $p_{\mathrm{CE}}=42/143\approx 0.294$. The results are unconditional in the sense that the properties of the different types of CIs (conditional and unconditional as described in Section 4.1) are averaged across all trial realizations, regardless of the stage the trial stops. The probability of stopping early for efficacy in Stage 1 is 0.308. Note that with *N* = 10^5^ trial replicates, the Monte Carlo standard error for the coverage is less than 0.0016.

**TABLE 3:** Simulation results showing the performance of various CIs with $p_{p}=21/134\approx 0.157$ and $p_{\mathrm{CE}}=42/143\approx 0.294$.

Type of CI	CI method	Coverage	Mean width (SD)	Consistency	P(L(X)>θ)	P(U(X)<θ)
Standard/naive	Wald test	0.945	0.203 (0.017)	0.989	0.029	0.026
Unconditional	Final	0.952	0.211 (0.019)	0.998	0.022	0.026
	Repeated	0.973	0.240 (0.063)	1.000	0.002	0.025
	Adjusted asymptotic	0.945	0.205 (0.020)	0.985	0.022	0.034
	Parametric bootstrap	0.926	0.210 (0.010)	0.988	0.056	0.018
Conditional	Final	0.954	0.584 (1.675)	0.732	0.024	0.022
	Restricted final	0.954	0.227 (0.039)	0.984	0.024	0.021
	Likelihood	0.988	0.630 (0.675)	0.693	0.002	0.010
	Penalized likelihood	0.988	0.271 (0.028)	0.992	0.002	0.010

*Note:* There were 10^5^ trial replicates. The probability of stopping at Stage 1 is 0.308. Note that if the final conditional and conditional likelihood CIs are constrained to lie in the interval (−1, 1) then the mean width (SD) becomes 0.390 (0.279) and 0.513 (0.420), respectively.

Starting with the coverage of the CIs, the standard CI has a very slight undercoverage, which is caused by two factors: (1) the distributional assumptions underlying the Wald CI are no longer met due to the stopping rule, and (2) the quality of the (asymptotic) normal approximation used for binomial outcomes. As expected, the final unconditional CI attains the nominal coverage of 95% (within Monte Carlo error). In contrast, the RCI has conservative coverage, which is driven by the trial replicates that stop early at Stage 1 (see Tables 4 and 5). The adjusted asymptotic CI has the same coverage as the standard CI. The parametric bootstrap CI has particularly low coverage of < 93% in this scenario.

Turning to the conditional estimators, both the conditional final CI and conditional restricted final CI attain the nominal coverage of 95% (with a very slight overcoverage). This is to be expected theoretically, since if a CI attains (or exceeds) the nominal coverage conditional on stopping at Stage 1 and conditional on continuing to Stage 2, then it will also attain (or exceed) the nominal coverage unconditionally (averaged over the two stopping possibilities). In contrast, the conditional likelihood and penalized likelihood have a notably conservative coverage, which is at least partly due to the choice of the conditional bootstrap procedure (using the bias‐corrected bootstrap, for example, would give different results but is out of scope of this paper).

Looking at the mean CI width, the CI methods with higher coverage compared with the standard CI also have a higher mean width. However, even though the parametric bootstrap CI has a lower coverage than the standard CI, it also has a slightly higher mean width. The mean widths of the unconditional final, adjusted asymptotic, and parametric bootstrap CIs are all within +4% of the mean width of the standard CI. In contrast, the RCI has a substantial increase of +17%, which is driven by the larger mean width conditional on stopping at Stage 1 (see Table 4). As for the conditional CIs, what is striking are the huge increases in mean width for the conditional final and conditional likelihood CIs, of +188% and +197%, respectively. These are also accompanied by very high standard deviations, reflecting a very high variability in the confidence limits. This is driven by very poor properties for these two methods when stopping at Stage 1 (see Table 4), especially when the observed test statistic is close to the stopping boundary. Such results have previously been noted in the literature [25, 26, 29]. In contrast, the conditional restricted final and penalized likelihood CIs have mean widths much closer to that of the standard CI, although there is still an increase of +12% and +33%, respectively. Again, this reflects the loss of information associated with conditioning on the stopping stage.

**TABLE 4:** Simulation results showing the performance of various CIs with $p_{p}=21/134\approx 0.157$ and $p_{\mathrm{CE}}=42/143\approx 0.294$, conditional on the trial stopping early at Stage 1.

Type of CI	CI method	Coverage	Mean width (SD)	Consistency	P(L(X)>θ)	P(U(X)<θ)
Standard/naive	Wald test	0.907	0.225 (0.010)	1.000	0.093	0.000
Unconditional	Final	0.930	0.234 (0.011)	1.000	0.070	0.000
	Repeated	0.995	0.334 (0.015)	1.000	0.005	0.000
	Adjusted asymptotic	0.930	0.232 (0.011)	1.000	0.070	0.000
	Parametric bootstrap	0.820	0.207 (0.010)	1.000	0.180	0.000
Conditional	Final	0.970	1.315 (2.884)	0.179	0.017	0.013
	Restricted final	0.970	0.243 (0.050)	0.997	0.017	0.013
	Likelihood	0.995	1.462 (0.689)	0.030	0.005	0.000
	Penalized likelihood	0.993	0.298 (0.017)	1.000	0.007	0.000

*Note:* There were 10^5^ trial replicates. The probability of stopping at Stage 1 is 0.308. Note that if the final conditional and conditional likelihood CIs are constrained to lie in the interval (−1, 1) then the mean width (SD) becomes 0.686 (0.351) and 1.087 (0.307), respectively.

In terms of consistency, the standard CI has consistency just below 99%, with instances of non‐consistent CIs being caused by the same reasons as for the undercoverage. The unconditional final CI has a coverage just below 100%, with the non‐consistency caused by the quality of the (asymptotic) normal approximation used for binomial outcomes, see also Lloyd (2021). The RCI is the only CI method with 100% consistency, as would be expected theoretically. Both the adjusted asymptotic and unconditional parametric bootstrap CIs have very similar consistency to the standard CI. For the conditional CIs, again it is striking how low the consistency is for the conditional final and conditional likelihood CIs, which is driven by how they are so wide (on average) that they often will include zero even when the null hypothesis of no treatment effect is rejected. In contrast, the conditional restricted final and penalized likelihood CIs have consistencies much closer to 100%, comparable to the consistency of the standard CI.

Finally, looking at the upper and lower coverages, these are approximately equal for the standard, unconditional final, conditional final, and conditional restricted final CIs. The upper coverage is (sometimes substantially) greater than the lower coverage for the RCI, adjusted asymptotic, conditional likelihood, and penalized likelihood CIs. The unconditional parametric bootstrap CI is the only method to have the lower coverage higher than the upper coverage.

**FIGURE 1:**
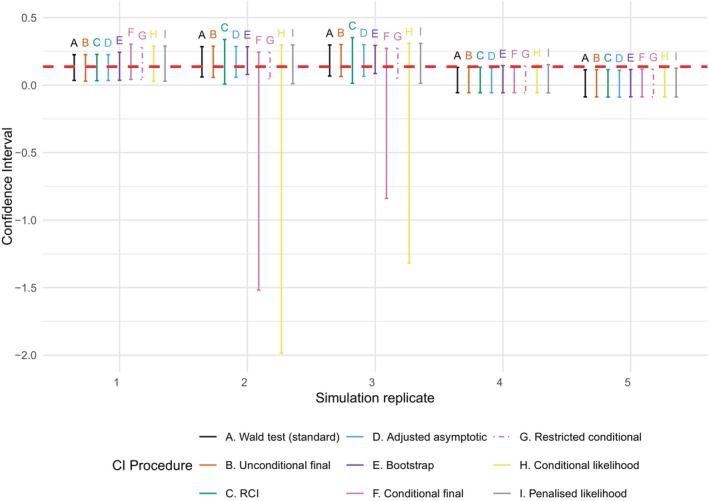
Confidence intervals from the first five simulation replicates of the simulation study. The horizontal red dashed line shows the true value of the treatment difference. RCI, repeated confidence interval.

In order to show more clearly the differences between the CIs in repeated realizations of the MUSEC trial, Figure 1 shows the CIs from the first five simulation replicates of the simulation study. Note that in Simulation Replicates 2 and 3, the trial stopped early at Stage 1 (and hence with rejection of the null hypothesis). In Simulation Replicates 1, 4, and 5, the trial continued to Stage 2, with rejection of the null hypothesis in Simulation Replicate 1.

Looking first at coverage (i.e., whether the CI contains the true value of the treatment difference, shown by the red horizontal line), in Simulation Replicates 1, 2, and 3, all CI methods contain the true value, whereas in Simulation Replicate 5, all CI methods do not contain the true value. However, in Simulation Replicate 4, we can see a discrepancy in the coverage, with the standard, unconditional final, RCI, and adjusted asymptotic CIs not containing the true value, whereas the other CIs do contain the true value. Similarly, in terms of consistency, in Simulation Replicates 2 and 3, the conditional final and conditional likelihood CIs contain zero, despite the null hypothesis of no treatment effect being rejected.

Finally, in terms of CI width, in the simulation replicates where the trial stopped early in Stage 1, it is striking how much wider both the conditional final and conditional likelihood CIs are compared to any of the others. Indeed, the lower bounds of the conditional likelihood CIs are below −1, which is also the case for the conditional final CI. In the trial replicates that continue to Stage 2, the CI widths are much more similar, although the conditional CIs are wider than the unconditional ones (as would be expected).

#### 4.2.2 Results Conditional on Stopping at Stage 1

Apart from the overall (unconditional) results, it is informative to also report results conditional on the stopping stage of the trial. Table 4 shows the simulation results conditional on stopping early at Stage 1, with the true success rates $\left(p_{p},p_{\mathrm{CE}}\right)$ on the two arms again equal to the overall observed means in the MUSEC trial. With a probability of early stopping of 0.308, these results are based on $3.08\times 10^{4}$ simulation replicates, giving a Monte Carlo standard error of less than 0.0028 for the coverage.

Conditional on stopping at Stage 1, the coverage of the standard CI is substantially below the nominal, at less than 91%. The coverage of the unconditional final and adjusted asymptotic CIs also decreases to 93%, while the parametric bootstrap has the largest drop to only 82%. In contrast, the RCI has a very conservative coverage of 99.5%. These results demonstrate that even if unconditionally the unconditional CIs may have near nominal coverage, the conditional coverage properties can be poor. In contrast, all of the conditional CI methods (as expected) have coverage above the nominal 95%, with the conditional final and restricted final CIs having conservative coverage of 97%, and the conditional likelihood and penalized likelihood CIs having a very conservative coverage comparable to the RCI.

In terms of mean CI width, similar patterns are seen as for the unconditional results. Some noticeable features are that the RCI has a substantially higher mean width than either the conditional restricted final or penalized likelihood CIs. The very large mean widths of the conditional final and conditional likelihood CIs are even more extreme conditional on stopping at Stage 1. This agrees with the literature [25, 26] that does not recommend the use of these two methods when a group sequential trial stops early for benefit. The very large mean widths also correspond with very low consistencies of the test decision (which is always to reject the null). In contrast, all other CI methods have 100% consistency (as would be expected in theory for the RCI and penalized likelihood CI) except for the restricted final CIs due to a few replicates having these as the empty set. Finally, all CI methods except for the conditional final and restricted final CIs have an upper coverage of zero.

#### 4.2.3 Conditional on Continuing to Stage 2

Table 5 shows the simulation results conditional on continuing to Stage 2, with the true success rates $\left(p_{p},p_{\mathrm{CE}}\right)$ on the two arms again equal to the overall observed means in the MUSEC trial. With a probability of continuing to Stage 2 of 69.2%, these results are based on $6.92\times 10^{4}$ simulation replicates, giving a Monte Carlo standard error of less than 0.0019 for the coverage.

**TABLE 5:** Simulation results showing the performance of various CIs with $p_{p}=21/134\approx 0.157$ and $p_{\mathrm{CE}}=42/143\approx 0.294$, conditional on the trial continuing to Stage 2.

Type of CI	CI method	Coverage	Mean width (SD)	Consistency	P(L(X)>θ)	P(U(X)<θ)
Standard/naive	Wald test	0.962	0.193 (0.008)	0.984	0.000	0.038
Unconditional	Final	0.962	0.200 (0.010)	0.998	0.000	0.038
	Repeated	0.963	0.198 (0.008)	1.000	0.000	0.036
	Adjusted asymptotic	0.951	0.193 (0.008)	0.978	0.000	0.049
	Parametric bootstrap	0.973	0.211 (0.009)	0.983	0.000	0.027
Conditional	Final	0.947	0.258 (0.040)	0.978	0.027	0.025
	Restricted final	0.947	0.220 (0.031)	0.978	0.027	0.025
	(Penalized) likelihood	0.985	0.258 (0.023)	0.989	0.000	0.015

*Note:* There were 10^5^ trial replicates. The probability of continuing to Stage 2 is 0.692.

Conditional on continuing to Stage 2, the standard CI and all the unconditional CIs now have slightly conservative coverage of around 96%–97% (with the exception of the adjusted asymptotic CI which achieves the nominal 95% coverage). In contrast, the coverage of conditional final and restricted final CIs is just below the nominal 95%. The conditional likelihood CI (which is the same as the penalized likelihood CI in this case) has the most conservative coverage of 98.5%. What is noticeable is that the magnitude of the relative increase in the mean widths of the conditional final and conditional (penalized) likelihood CIs compared to the standard CI is much lower compared to the relative increase conditional on early stopping at Stage 1.

In terms of consistency, there is a drop of up to around 2% (compared with the 100% consistency conditional on stopping at Stage 1) for the standard CI, conditional restricted final, and all unconditional CIs, with the exception of the RCI which maintains 100% consistency (as expected theoretically) and the unconditional final CI which has almost 100% consistency (with inconsistency again caused by the normal approximation). Meanwhile, the consistency of the conditional final and conditional likelihood CIs is around 98%–99%, compared with only 18% and 3% consistency, respectively, conditional on stopping at Stage 1. Finally, all CI methods except for the conditional final and restricted final CIs now have a *lower* coverage of zero.

If however we run the simulations with a higher true success rates for $p_{\mathrm{CE}}$, that is, $p_{\mathrm{CE}}=42/143+0.08\approx 0.374$ so that the probability of continuing to Stage 2 is only 0.239, the coverage results for the standard and unconditional CIs conditional on continuing to Stage 2 look rather different. Table 6 shows these simulation results, with the Monte Carlo standard error for the coverage and consistency results less than 0.0032.

**TABLE 6:** Simulation results showing the performance of various CIs with $p_{p}=21/134\approx 0.157$ and $p_{\mathrm{CE}}=42/143+0.08\approx 0.374$, conditional on continuing to Stage 2.

Type of CI	CI method	Coverage	Mean width (SD)	Consistency	P(L(X)>θ)	P(U(X)<θ)
Standard/naive	Wald test	0.897	0.202 (0.007)	0.994	0.000	0.103
Unconditional	Final	0.902	0.218 (0.012)	0.997	0.000	0.098
	Repeated	0.910	0.209 (0.007)	1.000	0.000	0.090
	Adjusted asymptotic	0.895	0.205 (0.007)	0.992	0.000	0.105
	Parametric bootstrap	0.959	0.221 (0.008)	0.995	0.000	0.041
Conditional	Final	0.945	0.310 (0.043)	0.989	0.031	0.024
	Restricted final	0.945	0.203 (0.055)	0.989	0.031	0.024
	(Penalized) likelihood	0.987	0.288 (0.019)	0.996	0.000	0.013

*Note:* There were 10^5^ trial replicates. The probability of continuing to Stage 2 is 0.239.

This time, the standard CI and all the unconditional CIs, including the RCI, now have coverage substantially less than the nominal 95%, ranging from 89% to 91%. The exception is the parametric bootstrap CI, which has a slightly conservative coverage (96%). In contrast, the coverage of conditional final and restricted final CIs remains just below the nominal 95%, while the coverage of the conditional (penalized) likelihood CI remains rather conservative (almost 99%). Again these results demonstrate that unconditional CIs can have poor coverage properties conditional on the stopping stage (regardless of whether the trial is stopped early or continues). Tables showing the simulation results unconditionally and conditional on early stopping at Stage 1 for this choice of values for $p_{p}$ and $p_{\mathrm{CE}}$ can be found in Appendix 3 (Tables A5 and A6).

#### 4.2.4 Simulation Results Across a Range of Values for $p_{\mathrm{CE}}$

**FIGURE 2:**
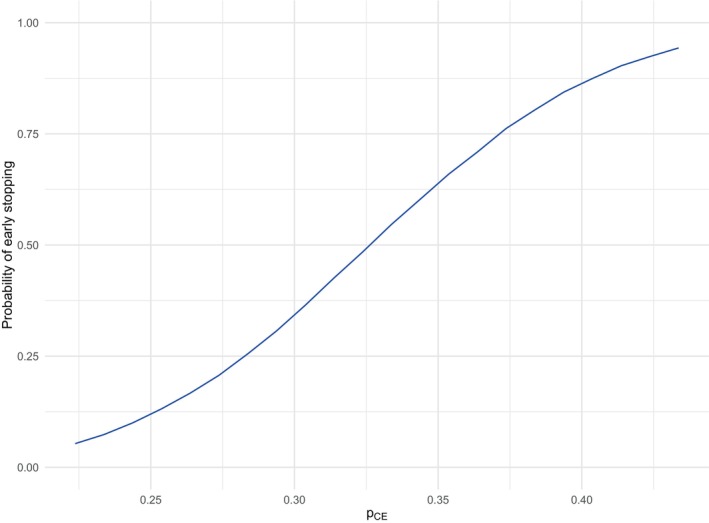
Probability of early stopping at Stage 1 as the value of $p_{\mathrm{CE}}$ varies from ${\hat{p}}_{\mathrm{CE}}-0.07\approx 0.224$ to ${\hat{p}}_{\mathrm{CE}}+0.14\approx 0.434$. The value of $p_{p}=21/134\approx 0.157$.

The results given in Tables 4, 5, 6 are only for a fixed value of $p_{\mathrm{CE}}$ and so we now explore the performance of the various CI methods across a range of values of $p_{\mathrm{CE}}$ from ${\hat{p}}_{\mathrm{CE}}-0.07\approx 0.224$ to ${\hat{p}}_{\mathrm{CE}}+0.14\approx 0.434$ (while keeping $p_{P}$ fixed), which corresponds to a probability of early stopping ranging from 0.05 to 0.94, as seen in Figure 2.

For each value of $p_{\mathrm{CE}}$ (22 in total), we ran *N* = 10^5^ trial replicates for each of the CI methods, except for the standard CI where we ran *N* = 10^6^ trial replicates in order to more accurately assess whether there was any undercoverage. For the CI methods requiring a bootstrap procedure (unconditional parametric bootstrap, conditional likelihood and conditional penalized likelihood), we again used *B* = 10^4^ bootstrap samples. We also constrained the final conditional and conditional likelihood CIs to lie in the interval (−1, 1).

**FIGURE 3:**
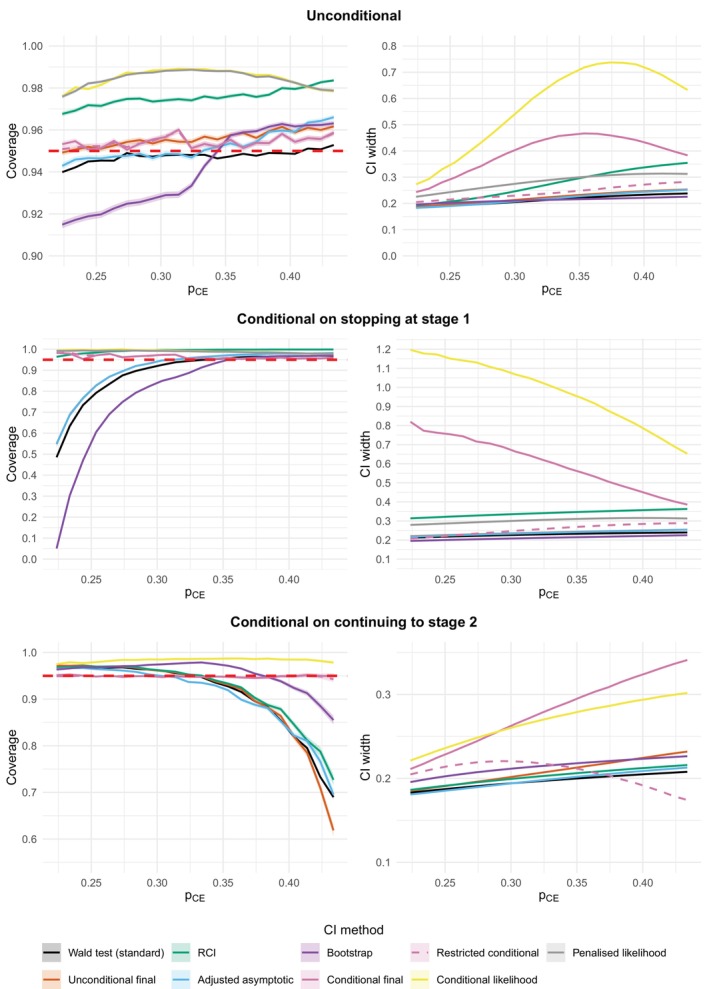
Coverage and CI width as the value of $p_{\mathrm{CE}}$ varies from ${\hat{p}}_{\mathrm{CE}}-0.07\approx 0.224$ to ${\hat{p}}_{\mathrm{CE}}+0.14\approx 0.434$. The value of $p_{p}=21/134\approx 0.157$. The shaded areas around the lines for the CI methods correspond to ±1.96 times the Monte Carlo standard error. The red dashed line denotes the nominal 95% coverage. For the unconditional coverage plot, the restricted conditional and conditional final lines are almost completely overlapping. For the coverage plot conditional on stopping at Stage 1, the unconditional final and adjusted asymptotic lines completely overlap, as do the restricted conditional and conditional final lines. The conditional likelihood and penalized likelihood lines are also very close together. For the CI width plot conditional on stopping at Stage 1, the unconditional final and adjusted asymptotic lines are almost completely overlapping. For the coverage plot conditional on continuing to Stage 2, the conditional final and restricted conditional lines are almost completely overlapping.

Figure 3 shows the coverage of the CI methods and the mean CI width as a function of $p_{\mathrm{CE}}$, split into three rows corresponding to unconditional, conditional on stopping at Stage 1 and conditional on continuing to Stage 2. The shaded areas around the lines for the CI methods correspond to ±1.96 times the Monte Carlo standard error. The red dashed line denotes the nominal 95% coverage.

Starting with the unconditional results, the coverage of the standard CI is (just) below the nominal 95% for most of the range of $p_{\mathrm{CE}}$ except for $p_{\mathrm{CE}}>0.40$ when it goes just above 95%. In contrast, the unconditional final CI has coverage (just) above the nominal 95% for the whole range of $p_{\mathrm{CE}}$, with the coverage becoming increasingly conservative (up to 96%) as $p_{\mathrm{CE}}$ increases. The RCI has rather conservative coverage for the whole range of $p_{\mathrm{CE}}$, with the coverage also becoming increasingly conservative (> 98%) as $p_{\mathrm{CE}}$ increases. The adjusted asymptotic CI has very similar undercoverage to the standard CI for $p_{\mathrm{CE}}<0.33$ but then has increasingly conservative coverage as $p_{\mathrm{CE}}$ increases above 0.33. The unconditional parametric bootstrap CI has rather low coverage (< 93%) for $p_{\mathrm{CE}}<0.32$ but quickly switches to having increasingly conservative coverage for $p_{\mathrm{CE}}>0.34$. These results demonstrate that whether a particular CI method has the correct coverage can strongly depend on the true (unknown) underlying parameter values. The conditional CIs all have conservative coverage, but the conditional final and conditional restricted final CIs have coverage close to the nominal (95%–96%). In contrast, the conditional likelihood and penalized likelihood CI have rather conservative coverage (98%–99%).

As for the unconditional mean width, the unconditional final, adjusted asymptotic and unconditional parametric bootstrap CIs all have similar mean width as the standard CI (within ±6%). The conditional restricted final CI has a slightly higher mean width than the standard CI (between 8% and 13%), with the penalized likelihood CI having a mean width between 23% and 33% higher than the standard CI. In contrast, the conditional final and conditional likelihood CI mean widths are dramatically larger than the standard CI, up to 106% and 203% larger, respectively. Finally, the RCI has a similar mean width as the standard CI for small values of $p_{\mathrm{CE}}$ but becomes substantially higher as $p_{\mathrm{CE}}$ increases (up to 43% larger).

Turning now to the coverage conditional on early stopping at Stage 1, for smaller values of $p_{\mathrm{CE}}$ the coverage of the standard CI, adjusted asymptotic CI, unconditional final and unconditional bootstrap CI is very low. The standard CI coverage can even be less than 50%. Note though that the lowest coverages are also achieved when there are the lowest probabilities of actually stopping at Stage 1. The coverage of the standard CI does go above the nominal 95% when $p_{\mathrm{CE}}>0.33$. In contrast, all the conditional CIs and the RCI have conservative coverage throughout the range of $p_{\mathrm{CE}}$, with the conditional final and restricted final CIs being (much) closer to the nominal level of coverage compared to the RCI, conditional likelihood and penalized likelihood CI.

The mean width of the CIs conditional on stopping at Stage 1 show similar patterns as for the unconditional results, except that the RCI now has substantially greater mean width than the standard CI across the range of $p_{\mathrm{CE}}$ values (consistently between 47% and 52% larger). Also, the extreme widths for the conditional final and conditional likelihood CIs are even more striking for smaller values of $p_{\mathrm{CE}}$. This reflects the results in the literature that these CIs have poor properties conditional on early stopping, with much better properties seen with the conditional restricted final and penalized likelihood CIs.

As for the coverage of the CIs conditional on continuing to Stage 2, the coverage of the standard, adjusted asymptotic, unconditional final and unconditional bootstrap CI is very low for larger values of $p_{\mathrm{CE}}$. The coverage of the standard CI can be below 70%. This time, the RCI is no longer conservative throughout the whole range of larger values of $p_{\mathrm{CE}}$, with also a very low coverage for larger values of $p_{\mathrm{CE}}$ (as low as 73%), which could in part be due to the positive bias of the MLE conditional on continuing to Stage 2. These lowest coverages are achieved when there are the lowest probabilities of actually continuing to Stage 2. The coverage of the standard CI is above the nominal 95% when $p_{\mathrm{CE}}<0.33$. The conditional likelihood CI (which is the same as the penalized likelihood CI) and the RCI again have conservative coverage throughout the range of $p_{\mathrm{CE}}$. This time the conditional final and restricted final CIs essentially match the nominal 95% level of coverage.

Finally, for the mean width of the CIs conditional on continuing to Stage 2, both the adjusted asymptotic CI and RCI have a similar mean width as the standard CI (within ±2% and up to 4% greater, respectively). Meanwhile, the unconditional final CI has an increasingly large mean width compared with the standard CI as $p_{\mathrm{CE}}$ increases (up to 12% larger). The unconditional bootstrap CI has a consistently higher mean width than the standard CI of between 7% and 9%. The mean width of the conditional restricted final CI has an interesting pattern, being up to 15% greater than the standard CI for $p_{\mathrm{CE}}<0.38$, but then having a smaller mean width than the standard CI for $p_{\mathrm{CE}}>0.38$, with even up to a 16% decrease. In contrast, the conditional final and conditional likelihood CI mean widths remain much larger than the standard CI, up to 64% and 46% larger, respectively. This implies that the penalized likelihood CI also has much larger widths than the standard CI conditional on continuing to Stage 2 (as it is equal to the conditional likelihood CI).

### 4.3 Discussion of Simulation Results

As noted when introducing the MUSEC trial case study, we use a GSD to illustrate the widest range of different types of adjusted CIs. Even though the simulation results are based on the specific two‐stage GSD used in the MUSEC trial, there are some observations we can make that are expected to hold in general for other types of ADs, based on generic theoretical considerations. We caution that these observations would still need to be systematically assessed for other types of ADs, which we return to in the Discussion (Section 6).

- There is often (but not always) the following tradeoff in metrics: a high(er) coverage implies high(er) mean CI width, and vice‐versa. The tradeoff between coverage and consistency is much less clear.
- While unconditional CIs can have good performance unconditionally, the conditional performance (especially in terms of coverage) may be poor.
- To guarantee conditional performance in terms of coverage, conditional CIs are a must. However, this can come at the price of (very) wide CIs.
- The simulations highlight the importance of looking at a wide(r) region of the parameter space, as some CI methods may perform well in some parts of the parameter space and not in others.

We can also make the following observations that are specific to the MUSEC trial (and other similar GSDs). Note that we have deliberately not claimed that one method is the “best” overall and should be used in the MUSEC trial context, as which method is “best” depends on the trial aims and relative importance of relevant metrics of interest (including coverage, consistency and CI width), see Section 5.

- The simulation results show that even in this relatively simpler setting of a GSD with two stages, there can be (very) large differences in the properties of the various adjusted CI methods.
- For conditional CIs, as has been discussed in the literature, the restricted conditional final CI is to be preferred to the conditional final CI, and the penalized likelihood CI is to be preferred to the conditional likelihood CI (particularly for trials that stop early at Stage 1).
- Some of the undercoverage and inconsistency is driven by the quality of the (asymptotic) normal approximation used. However, this is not unique to ADs, with such issues also seen for CIs for binary endpoints in fixed trial designs [21].
- The only CI methods that guarantee consistency in all cases in this trial context are the RCI and the penalized likelihood CI (when stopping at Stage 1).
- More “extreme” conditional results can be seen as the probability of stopping at that stage gets closer to zero or one.

## 5 Guidance: Best Practice for CIs in ADs

In this Section, we give guidance on the choice and reporting of CIs for ADs. This builds on the relevant parts of the FDA guidance for ADs [10] and the ACE guidance [2, 11], and closely follows the guidance for best practices for point estimation in ADs given by Robertson et al. [8, 9]. The choice of CIs should be considered throughout an adaptive trial, from the planning stage to the final reporting and interpretation of the results. Indeed, the design and analysis of an adaptive trial are closely linked, and should ideally go hand‐in‐hand. In what follows, our main focus is on the confirmatory setting where analyses are fully pre‐specified, but some of the principles can also apply to more exploratory settings (e.g., the CONSORT Dose‐Finding Extension guidelines mention the reporting of CIs [30]).

### 5.1 Planning Stage

The context, aims, and design of an adaptive trial should all inform the analysis strategy used, including the choice of CIs. These decisions are not only for trial statisticians but should also be discussed with other trial stakeholders to ensure consistency with what they want to achieve. First, it is necessary to decide on what exactly is to be estimated (i.e., the estimands of interest [28]). Second, the desired characteristics of potential CIs should be decided. Some key considerations are as follows:

- *Conditional* versus *unconditional perspective*: The choice of whether to look at the conditional or unconditional properties of CIs will depend on the trial design. For example, in a drop‐the‐losers trial where only a single treatment is selected for analysis in the final stage, a conditional perspective reflects the primary interest being in estimating the treatment effect of the selected treatment. On the other hand, for group sequential trials, the unconditional perspective is recognized as being an important consideration [9]. As seen in the simulation study in Section 4, the conditional properties of unconditional CIs can be poor, while the conditional CIs can have (much) larger widths than the unconditional CIs. A general framework of viewing the question of conditional versus unconditional inference is provided by Marschner [31]. Rather than advocating for or against unconditional inference over conditional inference in general, the framework allows for the exploration of the extent to which conditional bias is likely to be present within a given sample (using meta‐analysis techniques).
- *Trade‐off in metrics*: As highlighted at the end of Section 4, typically there will be a trade‐off between the coverage and the width of CI methods. In terms of interpretability/communication, consistency with the test decision is key. Depending on the context and aims of the trial, different relative importance may be given to the other criteria. For example, in a phase II trial where a precise estimate of the treatment effect is needed to inform a follow‐up confirmatory study, the CI width may be of greater concern, whereas in a definitive Phase III trial more emphasis may be placed on having the correct coverage to satisfy regulatory concerns. We are not aware of clear proposals in the literature on how to combine these different metrics into one overall criterion (as opposed to, say, the mean squared error [MSE] for point estimators).
- *Link with point estimation*: As highlighted in Sections 3 and 4, all of the CI methods (apart from the RCI) have a natural associated point estimator. Hence, the considerations and guidance around the choice of point estimators given in Robertson et al. [8, 9] can also play a role in the choice of the CI method. Ideally, these choices should go hand‐in‐hand to avoid the (rare) situations where the chosen point estimator lies outside the chosen CI.

In trials with multiple outcomes (e.g., primary and secondary outcomes), there may be different criteria and hence CIs needed for different outcomes. As well, in some trial settings such as multi‐arm trials where more than one arm can reach the final stage, the CI of each arm's comparison with control could be considered separately, but there may also be interest in calculating, for example, the simultaneous coverage across all arms that are selected. Once the criteria for assessing CIs have been decided, the next step is to find potential CIs that can be used for the trial design in question. Part I of this paper series is a starting point to find the relevant methodological literature.

For certain (more common) types of ADs, such as GSDs, a review of the literature may be sufficient to compare the different types of adjusted CIs. Otherwise, we would recommend conducting simulations to explore the properties of potential CIs given the AD. It is important to assess the CIs across a range of plausible parameter values and design scenarios, taking into account factors such as the probability of early stopping. The simulation‐based approach can also be used when there are no proposed alternatives to the standard CI for the trial design under consideration. Even in this setting, we would still encourage an exploration of the properties of the standard CI. If there is undercoverage or inconsistencies with the hypothesis test decision (for example), then this can impact how the results of the trial are reported (see Section 5.4). Exploring a bootstrap approach as an alternative to the standard CI may be an option in such a scenario.

### 5.2 Pre‐Specification of Analyses

The statistical analysis plan (SAP) and health economic analysis plan (HEAP) should include a description of the CIs that are planned to be used when reporting the results of the trial, and a justification of the choice of CIs based on the investigations conducted during the planning stage. This reflects FDA guidance, which states that there should be “prespecification of the statistical methods that will be used to […] estimate treatment effects…” [10]. The trial statistician and health economist should work together to develop plans that are complementary to both their analyses.

In settings where multiple adjusted CIs are available and are of interest, one CI should be designated the “primary” CI for the final reporting of results, with the others included as sensitivity or supplementary analyses (depending on the estimand of interest). This is to aid clarity in the interpretation of the trial results, and to avoid “cherry‐picking” the most favorable CI after observing the trial results. We have avoided making general recommendations on which CI method to use in practice because this depends on the context and goals of the trial, as well as the type of AD in question. In addition, given that CIs for ADs is an ongoing research area, there is a risk that any such recommendations may become outdated.

### 5.3 Data Monitoring Committees (DMCs)

When presenting interim results to DMCs, the choice of CIs should also be considered. For example, for GSDs the RCI has been suggested as a useful data monitoring tool [32].

### 5.4 Reporting Results for a Completed Trial

When reporting results for a completed adaptive trial, there should be a clear description of the “statistical methods used to estimate measures of treatment effects” [2]. Hence, it should be made clear what CI method is used, along with any underlying assumptions made in their calculation (e.g., being conditional on the observed stopping time). These discussions would naturally link back to the planning stage literature review and/or simulations (which could potentially be updated in light of the trial results and any unplanned adaptations that took place). For example, if the potential undercoverage of the standard CI is likely to be negligible, this would be a reassuring statement to make. On the other hand, in a setting where no adjusted CIs currently exist in the literature and there is the potential for undercoverage or any other performance issue of the standard CI, a statement flagging up this potential concern would allow appropriate caution to be taken when using the CI to inform clinical or policy decisions, future studies or meta‐analyses. As discussed in Section 5.2, it should be specified in advance (i.e., in the SAP for a confirmatory study) which CI will be used for the primary analysis and which (if any) CI(s) will be used as a sensitivity analysis.

## 6 Discussion

There is a growing body of methodological literature proposing various adjusted CI methods for a wide variety of ADs, with GSDs in particular having a large number of different options, as illustrated in our case study and simulation results. However, in our experience, there is at best limited uptake of adjusted CIs in practice, with many adaptive trials continuing to only report the standard CI.

It is our hope that this paper series will encourage the increased use and reporting of adjusted CIs in practice for ADs wherever possible. As described in our guidance in Section 5, estimation issues should be considered in the design stage of an adaptive trial. The estimation strategy should take the design of the trial into account, which motivates the use of adjusted CIs. In terms of trial reporting, statements about the potential undercoverage (for example) of the reported CIs can indicate where more care is needed in the interpretation of the results and the use of these CIs for further research.

For future research, it would be helpful to have stronger guidance on how to choose CIs in practice for a given AD type, especially in terms of proposals around how to appropriately combine different metrics/performance measures of interest. In addition, the case study and simulation results are based on a specific two‐stage GSD with early stopping for efficacy only. Although we have drawn some more general conclusions from these results in Section 4.3, it would be useful to systematically assess the performance of different methods for adjusted CIs for other types of ADs (and indeed other GSD stopping boundaries). Another important area of research is to explore how adjusted CIs perform when the statistical model used adjusts for randomization (e.g., stratification or minimization) factors or/and prognostic factors in line with methodological [33] and regulatory guidance [34, 35]. Finally, there is also the need for the further development of user‐friendly software and code for calculation of adjusted CIs in practice and to aid in simulations.

## Conflicts of Interest

The authors declare no conflicts of interest.

## Data Availability

R code to reproduce all the findings of this study is available at https://github.com/dsrobertson/CIs_for_ADs. For the purpose of open access, the author has applied a Creative Commons Attribution (CC BY) license to any Author Accepted Manuscript version arising.

## Appendix 1 Calculations of CIs—Additional Details

### Definition of the Information at Stages 1 and 2

At stage *k* (*k* = *1*,*2*), let ${\tilde{p}}_{k}$ denote the pooled estimate of the mean overall success probability, that is, the total number of observed successes divided by the total number of subjects. Then the observed information *I*_*k*_ is given by

$$I_{k}=\frac{1}{{\tilde{p}}_{k}\left(1-{\tilde{p}}_{k}\right)\left(1⁄n_{0k}+1⁄n_{\mathrm{CE}k}\right)}$$

where *n*_0*k*_ and *n*_CE*k*_ are the number of subjects on the placebo and CE arms, respectively, at stage *k*.

### Final Unconditional CI

The formula for the *p*‐value function for stopping stage *T* = 2 (with observed second‐stage test statistic $Z_{2}=z_{2}$) is as follows:

$$P\left(\theta \right)=\int_{-\infty }^{e_{1}}\int_{z_{2}}^{\infty }f_{2}\left(\left(x_{1}x_{2}\right),\left(\theta \sqrt{I_{1}}\theta \sqrt{I_{2}}\right),\left(\begin{array}{ll} 1 & \sqrt{I_{1}/I_{2}}\\ \sqrt{I_{1}/I_{2}} & 1 \end{array}\right)\right)dx_{2}dx_{1}$$

where $f_{2}\left(\left(x_{1}x_{2}\right),\mu ,\sum \right)$ is the density of a bivariate normal distribution with mean vector μ and covariance matrix ∑ evaluated at the vector $\left(x_{1},x_{2}\right)$.

### Adjusted Asymptotic CI

The functions $\hat{\mu }\left(\hat{\theta }\right)$ and $\hat{\sigma }\left(\hat{\theta }\right)$ are as follows:

$$\hat{\mu }\left(\hat{\theta }\right)=E\left(\hat{\theta }\right)-\hat{\theta }\;E\left(\sqrt{I_{T}}\right)$$

$$\hat{\sigma }\left(\hat{\theta }\right)=E\left(Z_{T}^{2}/I_{T}\right)-2\hat{\theta }\;E\left(Z_{T}\right)+{\hat{\theta }}^{2}\;E\left(I_{T}\right)-\hat{\mu }{\left(\hat{\theta }\right)}^{2}$$

### Unconditional Parametric Bootstrap Procedure

1. Given the end‐of‐trial response rate estimates ${\hat{p}}_{\mathrm{CE}}$ and ${\hat{p}}_{P}$, generate *B* bootstrap Stage 1 samples $S_{1,\mathrm{CE}}^{\left(1\right)},\ldots ,S_{1,\mathrm{CE}}^{\left(B\right)}$ and $S_{1,P}^{\left(1\right)},\ldots ,S_{1,P}^{\left(B\right)}$, where $S_{1,\mathrm{CE}}^{\left(b\right)}∼\mathrm{Bin}\left(n_{1,\mathrm{CE}},{\hat{p}}_{\mathrm{CE}}\right)$ and $S_{1,P}^{\left(b\right)}∼\mathrm{Bin}\left(n_{1,P},{\hat{p}}_{P}\right)$ represent the bootstrap number of successes on the CE and placebo arm, respectively, at the end of Stage 1 for *b* = 1, …, *B*. Here $n_{1,\mathrm{CE}}$ and $n_{1,P}$ are the Stage 1 sample sizes for the CE and placebo arm, respectively.
2. For *b* = 1, …, *B* calculate the bootstrap standardized Stage 1 test statistic $Z_{1}^{\left(b\right)}$ from the bootstrap values $S_{1,\mathrm{CE}}^{\left(b\right)}$ and $S_{1,P}^{\left(b\right)}$.
  - If $Z_{1}^{\left(b\right)}>e_{1}$ then the bootstrap MLE ${\hat{\theta }}^{\left(b\right)}$ is set equal to the Stage 1 MLE, that is,
    $${\hat{\theta }}^{\left(b\right)}=S_{1,\mathrm{CE}}^{\left(b\right)}/n_{1,\mathrm{CE}}-S_{1,P}^{\left(b\right)}/n_{1,P}$$
  - Otherwise, generate a bootstrap Stage 2 sample $S_{2,\mathrm{CE}}^{\left(b\right)}$ and $S_{2,P}^{\left(b\right)}$, where $S_{2,\mathrm{CE}}^{\left(b\right)}∼\mathrm{Bin}\left(n_{\mathrm{CE}}-n_{1,\mathrm{CE}},{\hat{p}}_{\mathrm{CE}}\right)$ and $S_{2,P}^{\left(b\right)}∼\mathrm{Bin}\left(n_{p}-n_{1,P},{\hat{p}}_{P}\right)$ represent the bootstrap number of successes on the CE and placebo arm, respectively, for Stage 2 only. Then the bootstrap MLE ${\hat{\theta }}^{\left(b\right)}$ is set equal to the overall MLE, that is,
    $${\hat{\theta }}^{\left(b\right)}=\left(S_{1,\mathrm{CE}}^{\left(b\right)}+S_{2,\mathrm{CE}}^{\left(b\right)}\;\right)/n_{\mathrm{CE}}-\left(S_{1,P}^{\left(b\right)}+S_{2,P}^{\left(b\right)}\right)/n_{P}$$
3. The bootstrap CI is then given by $\left(q_{\alpha /2},q_{1-\alpha /2}\;\right)$ where $q_{\alpha /2}$ and $q_{1-\alpha /2}$ are the α/2 and (1−α/2) quantiles, respectively, of the set ${\hat{\theta }}^{\left(1\right)},\ldots ,{\hat{\theta }}^{\left(B\right)}$.

### Randomization‐Based CI

The bootstrap procedure is defined as follows [24]. Suppose a group sequential trial has been completed, and let **X** denote the set of all observed patient outcomes. Let the vector **A** denote the set of all possible allocations of patients to treatments (given the randomization procedure used). Let $a_{i}$ denote a potential allocation of patients to treatments and $a^{*}$ denote the actual allocation used in the trial. Then let *F*(**X**, $a_{i}$) be the measure of strength of evidence against the null hypothesis. The adjusted *p*‐value is then

$$p_{\text{adjusted}}=\sum_{a_{i}\mathrm{\epsilon A}}I\left\{F\left(X,a_{i}\right)\geq F\left(X,a^{*}\right)\right\}$$

For the measure of strength of evidence against the null hypothesis, we use stagewise ordering of the sample space (like for the unconditional final CI). The randomization‐based CI is based on the adjusted *p*‐value as follows:

$$\left[\;\Phi^{-1}\left(1-p_{\text{adjusted}}\right)\pm \Phi^{-1}\left(1-\alpha /2\right)\;\right]\;\sqrt{{\hat{p}}_{\mathrm{CE}}\left(1-{\hat{p}}_{\mathrm{CE}}\right)/n_{\mathrm{CE}}+{\hat{p}}_{P}\left(1-{\hat{p}}_{P}\right)/n_{p}}$$

The associated point estimator for this CI is then

$$\Phi^{-1}\left(1-p_{\text{adjusted}}\right)\sqrt{{\hat{p}}_{\mathrm{CE}}\left(1-{\hat{p}}_{\mathrm{CE}}\right)/n_{\mathrm{CE}}+{\hat{p}}_{P}\left(1-{\hat{p}}_{P}\right)/n_{p}}$$

### Conditional Final CI

For a trial continuing to Stage 2 (so T=2), the lower and upper and bounds for the CI, ${\hat{\theta }}_{l}$ and ${\hat{\theta }}_{u}$, are the solutions to the equations

$$\alpha /2=\int_{{\hat{\theta }}_{\mathrm{obs}}}^{\infty }f\left(\hat{\theta }|{\hat{\theta }}_{l},T=2\right)d\hat{\theta }\;\text{and}\;1-\alpha /2=\int_{{\hat{\theta }}_{\mathrm{obs}}}^{\infty }f\left(\hat{\theta }|{\hat{\theta }}_{u},T=2\right)d\hat{\theta }$$

where $f\left(\hat{\theta }\;|\;\theta ,T=2\right)$ is the conditional density of the MLE (conditional on continuing to Stage 2):

$$f\left(\hat{\theta }\;|\;\theta ,T=2\right)=\frac{1-\Phi \left(\frac{e_{1}/\sqrt{I_{1}}-\hat{\theta }}{1/I_{1}-1/I_{2}}\right)}{1-\Phi \left(e_{1}-\theta \sqrt{I_{1}}\right)}\frac{\exp\left[-\frac{I_{2}}{2}{\left(\hat{\theta }-\theta \right)}^{2}\right]}{\sqrt{2\pi /I_{2}}}$$

The associated point estimator is the conditional MUE, ${\hat{\theta }}_{\text{CMUE}}$, which is the solution to the following equation:

$$0.5=\int_{{\hat{\theta }}_{\mathrm{obs}}}^{\infty }f\left(\hat{\theta }|{\hat{\theta }}_{\text{CMUE}},T=2\right)d\hat{\theta }$$

### Bootstrap Procedure for the Conditional Likelihood CI (Conditional on Continuing to Stage 2)

1. Set *b* = 1.
2. Given the end‐of‐trial response rate estimates ${\hat{p}}_{\mathrm{CE}}$ and ${\hat{p}}_{P}$, generate bootstrap Stage 1 sample $S_{1,\mathrm{CE}}^{\left(b\right)}$ and $S_{1,P}^{\left(b\right)}$, where $S_{1,\mathrm{CE}}^{\left(b\right)}∼\mathrm{Bin}\left(n_{1,\mathrm{CE}},{\hat{p}}_{\mathrm{CE}}\right)$ and $S_{1,P}^{\left(b\right)}∼\mathrm{Bin}\left(n_{1,P},{\hat{p}}_{P}\right)$.
3. Calculate the bootstrap standardized Stage 1 test statistic $Z_{1}^{\left(b\right)}$ from the bootstrap values $S_{1,\mathrm{CE}}^{\left(b\right)}$ and $S_{1,P}^{\left(b\right)}$.
  - If $Z_{1}^{\left(b\right)}>e_{1}$, go back to Step 2.
  - Otherwise, generate a bootstrap Stage 2 sample $S_{2,\mathrm{CE}}^{\left(b\right)}$ and $S_{2,P}^{\left(b\right)}$, where $S_{2,\mathrm{CE}}^{\left(b\right)}∼\mathrm{Bin}\left(n_{\mathrm{CE}}-n_{1,\mathrm{CE}},{\hat{p}}_{\mathrm{CE}}\right)$ and $S_{2,P}^{\left(b\right)}∼\mathrm{Bin}\left(n_{p}-n_{1,P},{\hat{p}}_{P}\right)$. The bootstrap conditional MLE ${\hat{\theta }}_{c}^{\left(b\right)}$ is calculated following the equation above, using the bootstrap values $S_{1,\mathrm{CE}}^{\left(b\right)},S_{2,\mathrm{CE}}^{\left(b\right)},S_{1,P}^{\left(b\right)}$, and $S_{2,P}^{\left(b\right)}$. Set *b* = *b +* 1 and go to Step 2.
4. The bootstrap CI is then given by $\left(q_{\alpha /2},q_{1-\alpha /2}\;\right)$ where $q_{\alpha /2}$ and $q_{1-\alpha /2}$ are the α/2 and (1−α/2) quantiles, respectively, of the set ${\hat{\theta }}_{c}^{\left(1\right)},\ldots ,{\hat{\theta }}_{c}^{\left(B\right)}$.

### Bootstrap Procedure for the Conditional Likelihood CI (Conditional on Early Stopping at Stage 1)

1. Set *b* = 1.
2. Given the Stage 1 success probability estimates ${\hat{p}}_{\mathrm{CE}}$ and ${\hat{p}}_{P}$, generate bootstrap Stage 1 sample $S_{1,\mathrm{CE}}^{\left(b\right)}$ and $S_{1,P}^{\left(b\right)}$, where $S_{1,\mathrm{CE}}^{\left(b\right)}∼\mathrm{Bin}\left(n_{1,\mathrm{CE}},{\hat{p}}_{\mathrm{CE}}\right)$ and $S_{1,P}^{\left(b\right)}∼\mathrm{Bin}\left(n_{1,P},{\hat{p}}_{P}\right)$.
3. Calculate the bootstrap standardized Stage 1 test statistic $Z_{1}^{\left(b\right)}$ from the bootstrap values $S_{1,\mathrm{CE}}^{\left(b\right)}$ and $S_{1,P}^{\left(b\right)}$.
  - If $Z_{1}^{\left(b\right)}>e_{1}$ then the bootstrap conditional MLE ${\hat{\theta }}_{c}^{\left(b\right)}$ is calculated following the equation above, using the bootstrap values $S_{1,\mathrm{CE}}^{\left(b\right)}$ and $S_{1,P}^{\left(b\right)}$. Set *b* = *b +* 1 and go to Step 2.
  - Otherwise, go back to Step 2.
4. The bootstrap CI is then given by $\left(q_{\alpha /2},q_{1-\alpha /2}\;\right)$ where $q_{\alpha /2}$ and $q_{1-\alpha /2}$ are the α/2 and (1−α/2) quantiles, respectively, of the set ${\hat{\theta }}_{c}^{\left(1\right)},\ldots ,{\hat{\theta }}_{c}^{\left(B\right)}$.

## Appendix 2 Case Study

Figure A1 gives a graphical representation of CIs (and associated point estimates) calculated using the observed data from the MUSEC trial.

Table A2 gives the values of the adjusted CIs (and associated point estimates) described in Section 3.1, calculated using the OBF stopping boundaries from the MUSEC trial and under the assumption that $Z_{1}\approx e_{1}$ and the trial stops at Stage 1. We assume the following Stage 1 data given in Table A1 to achieve this (where the total number of subjects on placebo and cannabis extract are the same as those observed in the MUSEC trial):

**TABLE A1:** Assumed trial data with O'Brien–Fleming efficacy stopping boundaries under the assumption that $Z_{1}\approx e_{1}$ and the trial stops at Stage 1.

	Interim analysis	
	Placebo	Cannabis extract
Number of patients with relief from muscle stiffness	30	51
Total number of subjects	97	101
Observed standardized test statistic	2.799	
O'Brien–Fleming stopping boundary for efficacy	2.797	
O'Brien–Fleming stopping boundary for futility	−∞	

For the methods requiring repeated sampling/simulation, we use *N* = 10^6^ trial replicates.

**TABLE A2:** Confidence intervals (and associated point estimates) calculated using the data given in Table A1 and the O'Brien‐Fleming efficacy stopping boundaries from the MUSEC trial.

Type of CI	CI method	Point estimate	95% two‐sided CI	CI width
Standard/naive	Wald test	0.196 (overall MLE)	(0.062, 0.330)	0.268
Unconditional	Final	0.196 (MUE)	(0.059, 0.333)	0.274
	Repeated	—	(0.000, 0.391)	0.391
	Adjusted asymptotic	0.195	(0.060, 0.329)	0.268
	Parametric bootstrap	0.203	(0.082, 0.327)	0.245
	Randomization‐based	0.202	(0.068, 0.336)	0.268
Conditional	Final	−16.28 (conditional MUE)	(−87.50, −0.398)	87.10
	Restricted final	−16.28 (conditional MUE)	∅	—
	Likelihood	−23.58 (conditional MLE)	(−3.27, 0.344)	3.610
	Penalized likelihood	0.001 (penalized MLE)	(0.008, 0.344)	0.336

*Note:* The symbol ∅ denotes the empty set.

Table A4 gives the values of the adjusted CIs (and associated point estimates) described in Section 3.1, calculated using the OBF stopping boundaries from the MUSEC trial and under the assumption that $Z_{2}\approx e_{2}$ and the trial continues to Stage 2. We assume the following trial data in Table A3 to achieve this (where the total number of subjects on placebo and cannabis extract at each stage are the same as those observed in the MUSEC trial):

**TABLE A3:** Assumed trial data with O'Brien–Fleming efficacy stopping boundaries under the assumption that $Z_{2}\approx e_{2}$ and the trial continues to Stage 2.

	Interim analysis		Final analysis	
	Placebo	Cannabis extract	Placebo	Cannabis extract
Number of patients with relief from muscle stiffness	30	45	48	68
Total number of subjects	97	101	134	143
Observed standardized test statistic	1.976		1.978	
O'Brien–Fleming stopping boundary for efficacy	2.797		1.977	
O'Brien–Fleming stopping boundary for futility	−∞		1.977	

For the methods requiring repeated sampling/simulation, we again use *N* = 10^6^ trial replicates.

**TABLE A4:** Confidence intervals (and associated point estimates) calculated using the data given in Table A3 and the O'Brien–Fleming efficacy stopping boundaries from the MUSEC trial.

Type of CI	CI method	Point estimate	95% two‐sided CI	CI width
Standard/naive	Wald test	0.117 (overall MLE)	(0.002, 0.233)	0.231
Unconditional	Final	0.117 (MUE)	(0.001, 0.233)	0.233
	Repeated	—	(0.000, 0.235)	0.235
	Adjusted asymptotic	0.116	(0.003, 0.230)	0.227
	Parametric bootstrap	0.121	(0.002, 0.255)	0.253
	Randomization‐based	0.121	(0.006, 0.236)	0.231
Conditional	Final	0.131 (conditional MUE)	(0.004, 0.286)	0.282
	Restricted final	0.131 (conditional MUE)	(0.004, 0.286)	0.282
	(Penalized) likelihood	0.135 (conditional MLE)	(−0.002, 0.287)	0.289

## Appendix 3 Additional Simulation Results

Simulations with the true success rates $\left(p_{p},p_{\mathrm{CE}}\right)$ given by $p_{p}=21/134\approx 0.157$ and $p_{\mathrm{CE}}=42/143+0.08\approx 0.374$. The probability of stopping early for efficacy in Stage 1 is 0.761.

### Overall (Unconditional Results)

**TABLE A5:** Simulation results showing the performance of various CIs with $p_{p}=21/134\approx 0.157$ and $p_{\mathrm{CE}}=42/143+0.08\approx 0.374$.

Type of CI	CI method	Coverage	Mean width (SE)	Consistency	P(L(X)>θ)	P(U(X)<θ)
Standard/naive	Wald test	0.948	0.227 (0.016)	0.999	0.028	0.025
Unconditional	Final	0.958	0.240 (0.015)	0.999	0.019	0.023
	Repeated	0.977	0.318 (0.062)	1.000	0.001	0.022
	Adjusted asymptotic	0.956	0.236 (0.019)	0.998	0.019	0.025
	Parametric bootstrap	0.961	0.218 (0.009)	0.999	0.029	0.010
Conditional	Final	0.954	0.789 (3.097)	0.587	0.020	0.026
	Restricted final	0.954	0.258 (0.054)	0.992	0.020	0.021
	Likelihood	0.988	0.856 (0.597)	0.351	0.009	0.003
	Penalized likelihood	0.986	0.308 (0.024)	0.999	0.011	0.003

*Note:* There were 10^5^ trial replicates. The probability of stopping at Stage 1 is 0.761. Note that if the final conditional and conditional likelihood CIs are constrained to lie in the interval (−1, 1) then the mean width (SD) becomes 0.560 (0.273) and 0.739 (0.420), respectively.

### Conditional on Stopping at Stage 1

**TABLE A6:** Simulation results showing the performance of various CIs with $p_{p}=21/134\approx 0.157$ and $p_{\mathrm{CE}}=42/143+0.08\approx 0.374$.

Type of CI	CI method	Coverage	Mean width (SE)	Consistency	P(L(X)>θ)	P(U(X)<θ)
Standard/naive	Wald test	0.964	0.235 (0.009)	1.000	0.036	0.000
Unconditional	Final	0.975	0.246 (0.009)	1.000	0.025	0.000
	Repeated	0.998	0.352 (0.013)	1.000	0.002	0.000
	Adjusted asymptotic	0.975	0.246 (0.009)	1.000	0.025	0.000
	Parametric bootstrap	0.961	0.218 (0.009)	1.000	0.039	0.000
Conditional	Final	0.957	0.940 (3.536)	0.461	0.017	0.026
	Restricted final	0.957	0.276 (0.039)	0.993	0.017	0.026
	Likelihood	0.988	1.033 (0.580)	0.149	0.012	0.000
	Penalized likelihood	0.986	0.314 (0.022)	1.000	0.014	0.000

*Note:* There were 10^5^ trial replicates. The probability of stopping at Stage 1 is 0.761. Note that if the final conditional and conditional likelihood CIs are constrained to lie in the interval (−1, 1) then the mean width (SD) becomes 0.507 (0.297) and 0.881 (0.383), respectively.
